# Telerehabilitation for aphasia – protocol of a pragmatic, exploratory, pilot randomized controlled trial

**DOI:** 10.1186/s13063-018-2588-5

**Published:** 2018-04-02

**Authors:** Hege Prag Øra, Melanie Kirmess, Marian C. Brady, Ingvild Elisabeth Winsnes, Silje Merethe Hansen, Frank Becker

**Affiliations:** 1 Sunnaas Rehabilitation Hospital, Bjørnemyrveien 11, 1450 Nesoddtangen, Norway; 2 Institute of Clinical Medicine, University of Oslo, PO Box 1171, Blindern, 0318 Oslo, Norway; 3 Department of Special Needs Education, University of Oslo, PO Box 1171, Blindern, 0318 Oslo, Norway; 4 Nursing, Midwifery and Allied Health Professions Research Unit, Glasgow Caledonian University, Cowcaddens Road, Glasgow, G40BA Scotland

**Keywords:** Aphasia, Telerehabilitation, Videoconference, RCT design, Stroke, Intensive therapy, Treatment efficacy

## Abstract

**Background:**

The Cochrane review on the effectiveness of speech and language therapy for aphasia following stroke suggests intensity of therapy is a key predictor for outcome. Current aphasia services cannot provide intervention at the intensity observed within trial contexts because of resource limitations. Telerehabilitation could widen access to speech-language pathologists (SLPs) in geographically remote contexts and reduce the time spent on travel by the therapist and patient. The current academic literature within this field is in its infancy, with few trials of speech and language therapy (SLT) delivered by videoconference. Our pilot randomized controlled trial (RCT) will explore feasibility aspects and effectiveness of telerehabilitation for aphasia in addition to standard SLT.

**Method/design:**

Our study is a pragmatic, exploratory, pilot randomized controlled trial, where participants will be randomized to a telerehabilitation group or a control group. Both groups receive standard SLT (usual care) but the telerehabilitation group receives an additional 5 h of telerehabilitation per week over 4 weeks through videoconference. This additional telerehabilitation focuses on spoken language with an emphasis on word naming. We aim to include 40 patients in each group, with inclusion criteria being aphasia any time post stroke. Participants will be assessed blindly at pre-randomization (baseline), and 4 weeks and 4 months after randomization. The primary endpoint is naming ability 3 months after the completed intervention, measured by the Norwegian Basic Aphasia Assessment (NGA) naming subtest. Secondary endpoints include other subtests of the NGA, the VAST (Verb and Sentence Test) subtest sentence production, Communicative Effectiveness Index (CETI) and the Stroke and Aphasia Quality of Life scale (SAQOL-39). Experiences of patients and SLPs with telerehabilitation are assessed using questionnaires and semi-structured interviews. Statistical between group comparisons will be in line with an intention-to-treat analysis.

**Discussion:**

This pilot RCT of intensive language training by videoconference will contribute new scientific evidence to the field of aphasia telerehabilitation. Here, we describe our trial which will explore the feasibility of telerehabilitation for aphasia as an intervention, our choice of primary and secondary outcome measures and proposed analyses. Our trial will provide information for the development and delivery of future definitive RCTs.

**Trial registration:**

ClinicalTrials.gov, ID: NCT02768922. Registered on 11 May 2016. Last updated on 17 November 2017.

**Electronic supplementary material:**

The online version of this article (10.1186/s13063-018-2588-5) contains supplementary material, which is available to authorized users.

## Background

Today, stroke stands as a major and leading cause of disability worldwide. Each year, one estimates that about 11,000 people in Norway acquire their first stroke [1]. In Norway, stroke is the most common cause of disability in the older population, with a stable incidence for the last decade due to better preventive treatment and acute management [1]. An aging European population in combination with an improvement in the survival rates of stroke is expected to put an increased burden on the health care system in the years to come.

One of the most devastating consequences of stroke is aphasia, a disturbance in language function which can affect the ability to speak and includes difficulties with speech production, auditory comprehension, as well as reading and writing. It affects one third of stroke survivors [2], where about 60% of the patients show consistent communication impairment 1 year post stroke [3]. Aphasia impacts on the rehabilitation process, affecting rehabilitation outcomes in a negative manner. The presence of aphasia following stroke is a negative predictor for return to workforce [4]. People with aphasia report significantly worse health-related quality of life (HRQL) than stroke survivors without aphasia [5]. Hence, aphasia affects stroke survivors’ social life by isolating them from social networks and limiting social participation [6]. Furthermore, aphasia impacts on significant others, describing third-party disability in family members [7]. Effective rehabilitation for aphasia is vital to recovery.

As the future holds an increasingly aged population, the urgency to develop evidence-based, cost-effective interventions to improve rehabilitation of people with aphasia is profound [8]. The aim of aphasia rehabilitation is an improvement in speech and language functions, where speech and language therapy (SLT) is considered to be “gold standard.” Several studies claim that intensive SLT improves outcomes in aphasia [9–11]. The updated Cochrane review from 2016 on the effects of SLT for aphasia following stroke, provides evidence of the effectiveness of SLT in terms of improved functional communication, reading, writing and expressive language compared to no access to therapy [8]. This meta-analysis furthermore concludes that there is some evidence that therapy at high intensity, high dose or over a longer period may be beneficial compared to lower intensity, lower dose or over a shorter period of time [8]. Today, a wide range of different forms of therapy exists, from constraint-induced language therapy (CILT) and functional-orientated therapy to phonological, semantic and cognitive-linguistic approaches. To date, the evidence for the best therapeutic approach, and details about timing, frequency, duration, intensity and dose, is still unclear for the overall aphasia population.

Access to SLT services, the recruitment and retention of speech-language pathologists (SLPs) remains a problem worldwide [12]. In rural countries like Norway, providing tailored aphasia rehabilitation of sufficient intensity and duration is a challenge. An early start to rehabilitation, meeting the Norwegian national guidelines of ≥ 5 h of SLT per week [13], seems not to be provided for reasons as the lack of SLPs and a limited capacity for aphasia services in the municipalities [14]. There is a unique opportunity to use telemedicine solutions for providing therapy. Telerehabilitation represents a future service delivery model for aphasia beyond the traditional gold standard of “face-to-face” treatment [15]. Telerehabilitation has been defined as rehabilitation services delivered via information and communication technology, and is categorized as rehabilitation services provided at a distance [16, 17]. It has the potential to widen the access to SLPs with promise of better and more equitable services. However, the academic literature on aphasia telerehabilitation is yet in its infancy, with substantial gaps in the scientific evidence of the feasibility and effectiveness of this therapy approach. This seems to be the case for stroke rehabilitation in general, as a Cochrane review of all types of telerehabilitation services for stroke has concluded that there is limited trials and evidence to guide practice [18]. Studies of the use of videoconference in aphasia interventions is especially lacking, with only one pilot RCT on the effectiveness of telerehabilitation by videoconference included in the published Cochrane review on aphasia following stroke [19]. Thus, the need to provide high-quality RCTs to contribute scientific evidence and knowledge to this relatively new field of aphasia research is a priority.

There are barriers and advantages with telerehabilitation activities in SLT as elaborated in a recent review [20]. A great advantage is higher availability of treatment facilitating shorter waiting lists and reducing potential delay of treatment. Telerehabilitation can reduce traveling time and costs for both patient and therapist. As treatment takes place in the patient’s home environment, the rehabilitation can be goal orientated toward the patient’s everyday life, with greater involvement of family and caregivers [20]. Telemedicine uses computer-based technology, which might enable the use of interactive computer-based therapy programs, videogames or social media [21]. Following stroke, significant fatigue and motor dysfunction can make traveling to receive therapy a challenging task. By receiving therapy in the home, patients with fatigue can focus their energies on aphasia rehabilitation rather than the journey to the rehabilitation centre [15]. Favorable patient satisfaction with telerehabilitation has been described [22, 23]. Despite these advantages, there are several barriers that hinder the routine implementation of telerehabilitation into a clinical setting. To date, we lack cost-benefit and cost-effectiveness analyses on telerehabilitation. There are ethical issues and threats to privacy and confidentiality to address. The lack of infrastructure, technical and/or personal support, funding of equipment and the lack of reimbursement are other issues to consider [20].

We previously conducted a feasibility study to explore the feasibility of delivering SLT by videoconference [24]. Tailored speech-language therapy was tested through videoconference in four patients with aphasia. Patient satisfaction and feasibility aspects were mapped through logs, questionnaires and semi-structured interviews. Aphasia rehabilitation via videoconference was found to be acceptable to both patient and therapist, and considered sustainable and feasible with regards to technical, logistic, patient and data safety aspects [24]. In this article we present the protocol of a pragmatic, exploratory, pilot randomized controlled trial (RCT) of standard SLT augmented by additional hours of SLT by videoconference compared to standard SLT alone. This pilot trial targets a larger, randomized sample size recruited across a number of sites, providing a better understanding of feasibility and new technical arrangements beyond our earlier feasibility study.

Due to ethical issues we have chosen a design were we compare the effects of aphasia telerehabilitation alongside standard SLT (usual care) with standard SLT only. To date, we have limited evidence of the effectiveness of telerehabilitation, which limits the application of SLT via videoconference as a treatment option.

### Objectives and aims

The objective of this pragmatic exploratory pilot RCT is to investigate the feasibility and effectiveness of speech and language training by videoconference given in addition to standard aphasia rehabilitation (usual care).

The primary and main aim of this study is to pilot the RCT of the clinical effectiveness of augmented speech-language therapy intervention for people with aphasia after stroke delivered by videoconference over 4 weeks (in addition to standard aphasia rehabilitation). We will explore whether augmented speech-language therapy by videoconference can improve word naming 4 months post randomization, when compared to standard rehabilitation (usual care).

Secondary aims of our pilot trial are to inform our intervention content, delivery, technology as well as software and training requirements. We will also gather data to enhance our understanding of anticipated recruitment, intervention adherence and dropout and future sample size calculations and our choice of outcomes. We also aim to explore the links between our intervention and naming, functional communication, quality of life and other language impairments than naming at 4 months post randomization, and whether it is sustainable and feasible with regards to ethical, technical, logistic, patient and data safety aspects.

## Methods/design

### Design

**Fig. 1:**
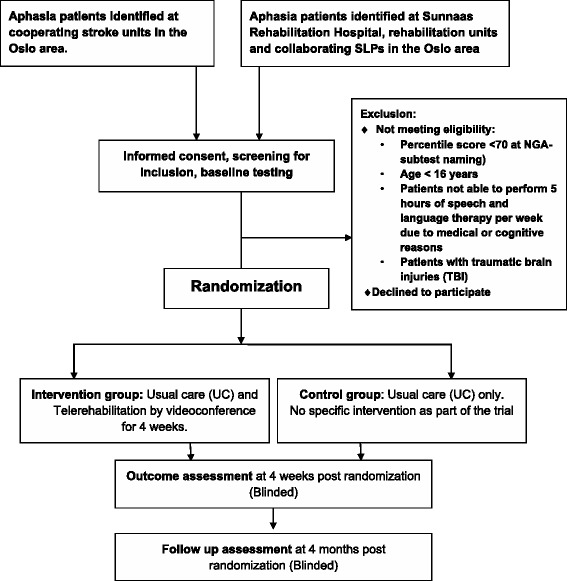
Flow diagram trial design

**Fig. 2:**
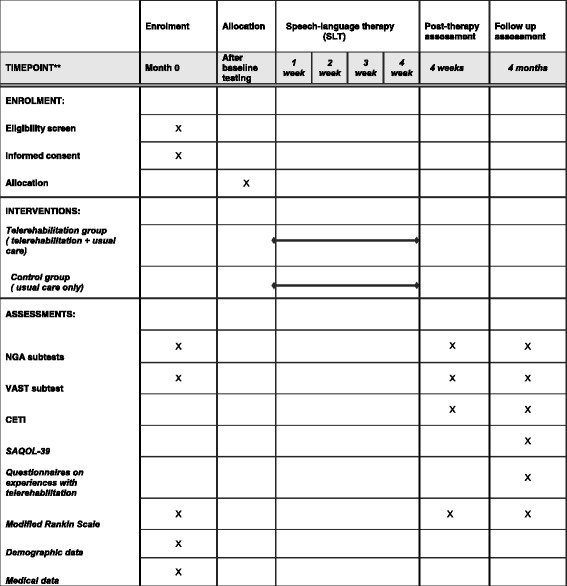
Trial schedule of enrollment, intervention and assessment (Standard Protocol Items: Recommendations for Interventional Trials (SPIRIT) Figure)

The study will use a pragmatic prospective RCT design (phase II exploratory trial), where outcomes will be compared for people with aphasia post stroke, randomly allocated to a telerehabilitation group or a control group. The telerehabilitation group will receive 5-h speech-language therapy via videoconference per week over 4 weeks, in addition to standard aphasia rehabilitation (usual care). The control group will receive standard speech-language therapy (usual care) only. The amount of standard aphasia rehabilitation will be logged for all participants. The patients will be assessed at inclusion and before randomization (baseline), and at 4 weeks and 4 months post randomization. Testing will be blinded. The protocol conforms to the Consolidated Standards of Reporting Trials (CONSORT) guidelines for pragmatic trials [25] and the guideline extensions for randomized pilot and feasibility trials [26]. The trial design is depicted by the flow diagram in Fig. 1. The timeline for study enrollment, intervention and assessment is illustrated in Fig. 2 (Standard Protocol Items: Recommendations for Interventional Trials (SPIRIT) Figure). A SPIRIT Checklist and CONSORT Checklist is included as Additional files 1 and 2.

### Ethics approval

The trial has been approved by the Norwegian Regional Committee South East for Medical and Health Research Ethics (reference number: 2015/2129, approval received in December 2015).

### Participants

We aim to recruit 80 participants with the diagnosis of aphasia following stroke. The patients will be recruited from the stroke units at Oslo University Hospital, Akershus University Hospital, Østfold Hospital and Bærum Hospital. Patients who are admitted for rehabilitation at Sunnaas Rehabilitation Hospital will also be included. In addition, patients or those on the waiting list at Sunnaas Rehabilitation Hospital will be invited to participate. We will also seek to recruit patients from other rehabilitation institutions, and in cooperation with speech-language pathologists in the region of Oslo, Østfold and Akershus. The recruitment of patients is limited to these areas due to geographical and practical aspects. Expressions of interest in participation will also be invited from members of the Aphasia Association of Norway (user organization).

### Inclusion criteria

Participants will be included if they meet the following criteria:

- Patients with aphasia following stroke (any time post stroke)
- Aphasia including naming impairment (percentile score of 70 or lower on the Norwegian Basic Aphasia Assessment (NGA) naming subtest)
- Norwegian as their main language

### Exclusion criteria

Participants will be excluded from the study if they meet any of the following criteria:

- Age below 16 years
- Patients who are unable to perform 5 h of speech-language therapy per week due to medical (including extensive hearing and/or vision impairment) or cognitive reasons
- Patients who score > 70 percentile score on the NGA naming subtest
- Patients with traumatic brain injury (TBI)

Patients with previous stroke are considered to meet inclusion. Patients will be included regardless of whether they are left or right handed. Regarding time post stroke, no limits were set in order to ensure a valuable sample size for this RCT, given the timeframe and geographical set up for the recruitment. This will result in a less homogenous sample, but increase ecological validity.

## Procedures

### Identification

Staff at recruitment sites will screen patients for eligibility, inform potential participants about the project and refer them to Sunnaas Rehabilitation Hospital for further investigation. Potential participants, who are patients at Sunnaas Rehabilitation Hospital, will receive information about the project and an invitation to take part in the trial. Members of the Aphasia Association of Norway will receive information about the study in a flyer and a brochure inviting them to contact the research team if they are interested in participating.

### Screening for eligibility, baseline assessment and recruitment

Detailed information about the project will be provided and the research investigator will seek informed consent before carrying out baseline testing. The Informed Consent Form and Information Sheet are accessible for people with aphasia. Relatives and caretakers will be carefully informed about participation. Eligibility will be established by an ambulatory visit (HPO, IEW) to the stroke unit, patient’s home or rehabilitation institution. Their eligibility will be confirmed by using clinical observations, medical information and language tests. The baseline assessment consists of the NGA subtests and the Verb and Sentence Test (VAST) subtest sentence production. If the potential participant is excluded, an explanation that the project is not suitable for the patient will be given. The number of those screened, but not included after screening, will be recorded along with the reason for exclusion. When included in the project, the participant’s demographic data will be collected including age, gender, relationship status and housing conditions. Relevant medical data, time post onset of stroke and type and location of stroke will be charted. The Modified Rankin Scale will describe the functional status of the patients, and be registered at each assessment time point (baseline, 4 weeks and 4 months; Fig. 1). Participants will be contacted up to three times for participation in outcome and follow-up assessment. No further data will be collected for those who discontinue study participation.

### Randomization and allocation concealment

If found to meet the inclusion criteria participants will be randomized directly after baseline assessment to the telerehabilitation group or to the control group. A web-based random sequence generator without limiting conditions will generate the randomization sequence. The randomization sequence list will be created in advance of recruitment by an experienced scientist who is not a member of the research team. Group allocation will be obtained by phone (once baseline data has been collected and recorded) from a member of staff who is not involved in the study at the outpatient clinic at Sunnaas Rehabilitation Hospital. Once the participant has been randomized, the research investigator will inform which group they have been allocated to. If the patient is included in the intervention group, further arrangement for the installation of the telerehabilitation equipment and training in the use of the computer will be addressed. The participant, relatives and/or caregivers will be given careful instructions on how to preserve allocation concealment in the 4-week control and follow-up testing, including instructions not to discuss treatment with the blinded SLPs.

### Blinding

Neither the participant nor the SLPs performing the therapy are blinded to treatment allocation due to the nature of the intervention. However, baseline assessment will be blinded as it will be performed before group allocation. The 4-week control and follow-up testing will be performed by external SLPs blinded to group allocation. All data collection sessions will be audio and video taped. In cases where allocation is inadvertently revealed by the patient, relatives and/or caregivers during conversation or by other means, a second SLP blinded to treatment allocation will re-score the test session using the recorded video tape.

## Outcome measures

### Primary endpoint

The primary endpoint of this study is naming ability 4 month post randomization, measured by the subtest “naming” in the NGA. The minimum difference of 8 percentile score is considered clinically significant. For the assessment of language functioning, the NGA [27] with the subtests comprehension, naming and repetition is included in the test battery. Percentile scores (i.e., percentile rank compared to the norm material of the NGA) will be used because they – for all severity levels of aphasia – mirror clinically relevant progression more accurately than raw scores.

### Key secondary endpoint(s)

Key secondary endpoints are other language functions 4 months post randomization measured by the NGA subtest “comprehension” and “repetition.” In addition, the VAST subtest sentence production [28], assesses the ability of verb and sentence production beyond words. Functional communication is assessed using the Communicative Effectiveness Index (CETI) [29]. These endpoints are scored/collected immediately after the intervention (4 weeks) and at the 4-month post randomization follow-up to detect any extended effect of the intervention.

### Other secondary endpoints

Quality of life will be assessed by using the Stroke and Aphasia Quality of Life scale (SAQOL-39) [30]. The SAQOL-39 is a quality of life scale developed specifically with regard to persons with aphasia. In addition, the experiences of patients, relatives and therapists with the telerehabilitation services and their participation within the trial will be gathered using questionnaires and semi-structured interviews with both SLPs and selected participants. Adverse events, including technical challenges, will be logged.

## Intervention

### Control group (usual care)

The control group will receive usual care provided by SLPs at the community level or in rehabilitation institutions. As usual care varies in the different municipalities and institutions, both in terms of type and frequency, the services provided for each participant will depend on the local resources available. The therapy may include face-to-face SLT in a single session or by group. The dose of therapy received from inclusion to follow-up 4 months post randomization will be recorded. Participants who are randomized to the control group will not receive any project specific intervention, but will, at the 4-month post-randomization follow-up, i.e., after their study participation, be referred for participation in an intensive language training program at Sunnaas Rehabilitation Hospital.

### Telerehabilitation group

As current scientific evidence favors intensity in aphasia interventions, our trial will offer a high-intensity therapy. Because there still is uncertainty about the best therapy approach within aphasia rehabilitation, no specific approach or theory has been chosen for our intervention beyond tasks that can enhance functional expressive communication. In the intervention group, the participants will receive augmented language training of 5 h a week over four consecutive weeks. This will be in addition to any usual SLT the participant is already receiving. The aim is to give 1 h SLT via videoconference per day, 5 days per week, but this can be adjusted to the participant’s timetable and rehabilitation schedule if necessary. Participants with ≥ 16 sessions over 32 days are considered to be per protocol. The SLT will be performed by an SLP using videoconference via the Internet from Sunnaas Rehabilitation Hospital to the laptop in the patient’s home, or rehabilitation/nursing ward. To secure treatment fidelity and replication for future studies and transparency of reporting, we have used the Template for intervention Description and Replication (TIDieR) Checklist and Guide (Additional file 3 [31]), to record and describe the intervention delivered by videoconference. The main features of the intervention using TIDier are illustrated in Table 1.

**Table 1:** The intervention illustrated by main features from the Template for intervention Description and Replication (TIDieR) Checklist and Guide

*Brief name:* Intensive speech and language therapy by videoconference	
*Why:* To improve expressive language function in patients with aphasia after stroke	
*What:* Intensive speech and language therapy with an emphasis on naming. The therapy will be tailored to the participant’s language impairment level and focus on expressive language and everyday communication. Material used in the training will include the Newcastle University Aphasia Therapy Resources (NUMA), a collection of SLP-made tasks for aphasia compiled as *Sareptas afasikrukke* and Lexia (computer-based training program). In addition, text and pictures from the Internet may also be used	
*Who provided:* Speech and language pathologists sited at Sunnaas Rehabilitation Hospital. Speech-language pathologists (SLPs) will receive training in how to give intervention by videoconference within the context of a clinical trial	
*How:* Using videoconference and remote control software to a laptop at the patient’s location	
*Where:* From Sunnaas Rehabilitation Hospital to the patient’s home or institution, e.g., rehabilitation ward or nursing home	
*When/How much:* The experimental intervention consists of 5 h of speech and language therapy a week, over 4 weeks (total dose of 20 h of therapy). Participants with ≥ 16 sessions over 32 days will be considered to be per protocol	

The SLT delivered by videoconference will be tailored to the participant’s language impairment. We will work on all language modalities; however, the intervention will have an emphasis on oral naming and speech production. As the therapy will be individualized, there will be a broad range of tasks including word production in “natural” sequences (e.g., weekdays and months), picture naming, discussion about familiar topics (e.g., hobbies and family) and conversations about a concrete subject, picture or situation. Since the main focus is expressive speech, material stimulating everyday communication will be the first choice and this will be tailored to the individual participant’s needs and goals. Material used in the intervention includes Norwegian versions of the Newcastle University Aphasia Therapy Resources (NUMA) [32–34], a collection of SLP-made tasks for aphasia compiled as *Sareptas afasikrukke* [35] and for some patients Lexia, a computer-based training program. In addition, text and pictures from the Internet, such as the easy to read newspaper *Klar Tale*, may be used if applicable. The SLPs in our trial will receive training in how to use the chosen material within the context of a clinical trial and computer software to provide the intervention by videoconference.

The broad range of material accessible to the intervention gives the therapist an optimal setting to tailor the therapy to the patient’s impairment level. The therapy will be in line with patient’s own goals, where a personalization of the therapy is considered a key of success with regards to compliance to protocol. Because the patients continue to receive ongoing care, we expect a wide range of variation of usual care with regards to type of therapy and the intensity with which it is given. Usual care will reflect upon rehabilitation resources available. To account for this expected heterogeneity, careful record of the usual care provided and accessed will be completed for all participants.

### Fidelity

For each participant in the intervention group, a report will be written. The report will describe the tailored therapy that each participant has received via videoconference. The content of the reports will be compared through regular fidelity reviews with the intervention description in the trial protocol.

## Technical solutions

As already tested in our feasibility study [24], we will use videoconference software and equipment installed at Sunnaas Rehabilitation Hospital and in study laptops given to the participants. The videoconference software is delivered by the “Norwegian Health Net (NHN),” a company owned by the Ministry of Health and Care services. Videoconference is provided through encrypted software which meets requirements with regards to data safety aspects, privacy and confidentiality. As the therapy sessions are live, there are no recordings or storage of video, sound or picture. The software we will use for the videoconference is Cisco Jabber/ Acano. In addition, the SLPs will also use the software called LogMeIn which allows the therapist to remotely control the participant’s computer, and has been shown to be a valuable tool in delivering therapy and assistance should technical problems arise [24].

Our current technical setup builds upon experience from the feasibility study “Language training straight home” [24]. In this former feasibility study some areas for improvement were identified which have been integrated in the present protocol. To maintain safety aspects a checklist has been implemented to be used at the start of each therapy session to control and adjust the patient’s physical environment. This is to ensure optimal training conditions, preserve accurate procedures in case of emergency and to accommodate privacy issues. When it comes to the technical aspects of delivering aphasia rehabilitation by videoconference, one expects to encounter technical challenges not revealed during the small feasibility study (*n* = 4). A separate log to map technical error has been developed and integrated into the protocol. Furthermore, the technical solutions tested in the feasibility study have been improved compared to the original technical setup. This includes a separate speaker to improve sound quality and a wide-angle web camera to enable the therapist to see the patient’s upper body in case alternative communication approaches, such as the use of body language and/or gestures, are needed.

## Sample size

No large RCTs have been carried out within the field of aphasia telerehabilitation, especially with the use of videoconference SLT. Previous studies have used interventions not applicable with the current protocol, and have been of varying quality and used small sample sizes. We were, therefore, unable to calculate an a prior accurate sample size. Thus, our pilot RCT will support a more accurate sample size estimate and to inform a definitive trial of the effect of intensive telerehabilitation for aphasia. We will investigate possible effects using the percentile score of the NGA naming subtest where we consider the minimal clinically meaningful effect to be an improvement of 8 percentile scores. Given a standard deviation of 18.17 (based on recently collected data from another project without telerehabilitation), 32 participants will be sufficient to detect a significant improvement within a group between time points using a pairwise comparison, with a significance level of 5 and a power of 80. This will enable the study to make some suggestions on effectivity. Considering possible dropouts, the study aims to include 40 participants in each group, with a total number of 80 subjects. We anticipate that results from this pilot RCT will yield data to inform a more accurate sample size calculation for future definitive RCTs.

## Statistical analysis

Data will be analyzed on an intention-to-treat basis. Descriptive statistics will be applied and the characteristics of the sample will be described. The trial is a longitudinal study with continuous repeated measurements. The data will be analyzed by using mixed models. A mixed model is preferred to the more traditional repeated measures analysis of variance (ANOVA) because of its advantages in the way in which it accommodates missing values. Data will be examined for differences over time and between groups. Subgroup analysis will be performed to explore factors considered to affect the outcome of the trial like differences in time since onset of stroke or demographic and stroke-related factors. Qualitative data collected from semi-structured interviews will be suitably coded for qualitative responses to be categorized and examined.

## Discussion

This protocol describes a pragmatic, pilot randomized controlled trial (phase II exploratory trial) that should make a valuable contribution to the field of aphasia telerehabilitation.

This trial’s objective is to investigate the feasibility and effectiveness of augmented SLT delivered via videoconference in addition to the standard SLT that the participants are already receiving. To our knowledge, there are no RCTs of this size that have explored the use of videoconferencing in providing an augmented, intensive SLT intervention for aphasia after stroke.

Our trial will look at the feasibility of scaling the intervention up for a larger trial and clinical implementation. Future studies may draw upon our findings if the trial is shown to be feasible, not harmful and indicates effects to be explored beyond this pilot. Our trial might offer some insight into videoconferencing as an effective mode of delivering speech and language interventions in the rehabilitation of people with aphasia, as we have chosen also to investigate clinically meaningful improvement. The trial does not meet sample size based on our assumptions for power calculation; however, large effect sizes might result in significant effects possibly influencing clinical practice. The main purpose of the study, however, is to contribute to the development of future fully powered definitive trials on the effectiveness of this therapy approach, also compared to – and not only in addition to – standard care.

A recent review of the effectiveness of SLT for post-stroke aphasia [8] suggests that intensity may be a key predictor for outcome. This trial investigates the feasibility of telerehabilitation as a supplement to increase the intensity of therapy within aphasia services with limited resources amongst a patient population with common mobility problems and fatigue within a geographically remote context. If telerehabilitation can be used within such a setting, our trial may demonstrate potential for change in future practice. Future studies examining SLT via videoconference as an alternative to usual care will be informed by this study. By shedding light on feasibility aspects through the data gathered using qualitative research methods and logs to map technical errors, we will expect to develop a greater understanding of SLT via videoconference.

When it comes to evaluate the feasibility of telerehabilitation for aphasia, broad participant inclusion criteria in this trial may be regarded as strength. Recent evidence suggests that there may be an interaction between timing post stroke and tolerance of high-intensity SLT [8]. By including participants at all stages of aphasia, from acute to chronic, the data will be more clinically representative for evaluating feasibility features, and of greater ecological validity to the population of people with aphasia. This trial is pragmatic in nature, and due to ecological validity, practicability and ethical issues, we have chosen to examine the effect of additional aphasia telerehabilitation alongside standard SLT in comparison to standard SLT only. As telerehabilitation is relatively new to the field of aphasia research and there is limited pre-existing evidence for the primary and secondary outcomes of our trial, it is considered unethical to prevent the patients from receiving usual care. Thus, an added benefit of this design is to reassure potential participants that randomization may augment their access to therapy and not restrict it.

### Trial status

Recruitment of patients started in May 2016, and 50 participants have been recruited and randomized into the trial.

## Additional files

Additional file 1: SPIRIT Checklist. (DOC 131 kb)

Additional file 2: CONSORT Checklist for Pilot and Feasibility Trials. (DOC 227 kb)

Additional file 3: TiDier Checklist. (DOCX 24 kb)

## Abbreviations

ANOVA: Analysis of variance
CETI: Communicative Effectiveness Index
CILT: Constraint-induced language therapy
CONSORT: Consolidated Standards of Reporting Trials
HRQL: Health-related quality of life
ITT: Intention-to-treat
NGA: Norwegian Basic Aphasia Assessment
NHN: Norwegian Health Net
NUMA: Newcastle University Aphasia Therapy Resources
RCT: Randomized control trial
SAQOL-39: Stroke and Aphasia Quality of Life Scale-39
SLP: Speech-language pathologists
SLT: Speech and language therapy
TIDieR: Template for Intervention Description and Replication
VAST: Verb and Sentence Test
