# Supplementary material for: Telerehabilitation for aphasia – protocol of a pragmatic, exploratory, pilot randomized controlled trial
Source: Trials. 2018 Apr 2;19:208. doi: 10.1186/s13063-018-2588-5 (PMC5880095; doi:10.1186/s13063-018-2588-5)
Supplement: Supplementary file 3 — TiDier Checklist. (DOCX 24 kb) [file 13063_2018_2588_MOESM3_ESM.docx]

**TIDieR checklist**

For the intervention given by videoconference, the template for Intervention description and replication (TiDieR) checklist and guide have been used. This to present a good description of the intervention and to ensure the possibility of replication for future studies.

| **TIDieR checklist** |  |
| --- | --- |
| **1.Brief name**  Provide the name or a phrase that describes the intervention | Intensive language and speech therapy (SLT) by videoconference |
| **2. Why**  Describe any rationale, theory, or goal of the elements essential to the intervention | The purpose of the SLT by videoconference is to increase the intensity of provided therapy and improve expressive language function with a focus on naming.  A mixed theoretical approach to our intervention following best practice has been chosen to enhance functional expressive communication. |
| **What:**  **3. Materials:** Describe any physical or informational materials used in the intervention, including those provided to participants  or used in intervention delivery or in training of intervention providers. Provide information on where the materials can be  accessed (such as online appendix, URL)  **4: Procedures**: Describe each of the procedures, activities, and/or processes used in the intervention, including any enabling or support activities | Since the main focus is expressive speech, material stimulating every day communication will be the first choice. The material includes the Newcastle University Aphasia Therapy Resources (NUMA), and a Norwegian collection of tasks developed by Speech-language pathologists (SLPs) for aphasia compiled as “Sareptas afasikrukke”. The materials can be bought at the Aphasia association of Norway (user organization). In addition we will use text and pictures from the internet, such as the easy to read newspaper Klar Tale (retrieved from http://www.klartale.no/). For some patients we will use Lexia, a computer based training program.  We will work on all language modalities; however the intervention will have an emphasis on oral naming and speech production. As the therapy will be individualized, there will be a broad range of tasks including word production in “natural” sequences (e.g. weekdays and months), picture naming, discussion about familiar topics (e.g. hobbies and family) and conversations about a concrete subject, picture or situation. Since the main focus is expressive speech, material stimulating everyday communication will be the first choice and this will be tailored to the individual participant. For patients with milder aphasia we will use crosswords, listen to or read a story and then retell it. Another task could be to tell about a hobby, occupation or other topics of interests |
| **5.** **Who provided:**  For each category of intervention provider (such as psychologist, nursing assistant), describe their expertise, background,  and any specific training given | Language and speech pathologists (SLPs) educated from Oslo university (2 SLPs) and the University of Bergen (1 SLP), employed at Sunnaas Hospital. Therapists have experience with patients with aphasia due to stroke. The SLPs will receive a 4 week program of approximately 100 hours of training in how to use the chosen therapy material within the context of a randomized controlled clinical trial and in the use of the computer software to provide the intervention by videoconference. |
| **6. How:**  Describe the modes of delivery (such as face to face or by some other mechanism, such as internet or telephone) of the intervention and whether it was provided individually or in a group | The telemedicine language training will be performed on a one-to-one patient to therapist basis via internet from Sunnaas Rehabilitation Hospital to a laptop at the patient’s location. We will use Cisco Jabber Video/Acano and the remote control software LogMeIn. Standard care therapy intervention will be given in individual 1-to-1 face-to-face sessions, and/or in face-to face group sessions. |
| **7. Where:**  Describe the type(s) of location(s) where the intervention occurred, including any necessary infrastructure or relevant features | In the patient’s home, or in a quite location/ therapy room at the institution e.g. rehabilitation ward or nursing home. |
| **8. When and How Much:** Describe the number of times the intervention was delivered and over what period of time including the number of sessions, their schedule, and their duration, intensity, or dose | Participants in the intervention group will receive one hour SLT via videoconference per day, five days per week, over 4 weeks. The delivery of the intervention will be adjusted to the participant’s timetable and rehabilitation schedule if necessary to meet the aim of 5 hours SLT via videoconference per week. Total dose of SLT via videoconference will be 20 hours by the end of the therapy intervention. The amount of sessions will be logged. Patients in the intervention group with at least 16 sessions of SLT via videoconference in 32 days will be regarded as “completed per protocol”.  All participants will also receive usual care provided by SLPs at the community level or in rehabilitation institutions. The rehabilitation services, both in terms of type and frequency, provided for each participant will depend on the local resources available. The usual care will be logged in each case. |
| **9. Tailoring:**  If the intervention was planned to be personalized, titrated or adapted, then describe what, why, when, and how | The SLT via videoconference will be personalized with regards to the level of impairment, linking functionally relevant words to for example hobbies and family. The impairment level is expected to be dynamic during the rehabilitation process, with increasing levels of difficulty of the therapy exercises. The intervention for each participant is done within a framework developed by the project group, and the speech-language pathologists in particular. The framework is described under checklist item 3 and 4.The intervention will be tailored to the individual participant’s needs and goals |
| **10*. Modifications:**  If the intervention was modified during the course of the study, describe the changes (what, why, when, and how) | Checklist is completed for a protocol, therefore not applicable. No changes are anticipated. |
| **How well**  **11. Planned**: If intervention adherence or fidelity was assessed, describe how and by whom, and if any strategies were used to maintain or improve fidelity, describe them  **12*. Actual**:  If intervention adherence or fidelity was assessed, describe the extent to which the intervention was delivered as  planned | To ensure intervention adherence, materials and language tasks were prepared in forehand. Intervention adherence will be assessed by referring to the documentation supporting each aphasia telerehabilitation course of therapy. We will also consider fidelity in a subset of interventions by reviewing the speech- language pathologists’ therapy charts and reports.  Checklist is completed for a protocol, therefore not applicable. |

*If checklist is completed for a protocol, these items are not relevant to protocol and cannot be described until study is complete.
